# Implementation of a palliative care intervention for patients with COPD – a mixed methods process evaluation of the COMPASSION study

**DOI:** 10.1186/s12904-022-01110-3

**Published:** 2022-12-07

**Authors:** Johanna M. C. Broese, Rianne M. J. J. van der Kleij, Els M. L. Verschuur, Huib A. M. Kerstjens, Yvonne Engels, Niels H. Chavannes

**Affiliations:** 1 Public Health & Primary care, Leiden University Medical Centre, Leiden, The Netherlands; 2 Lung Alliance Netherlands, Amersfoort, The Netherlands; 3 Respiratory Medicine & Tuberculosis, University Medical Centre Groningen, Groningen, The Netherlands; 4 Anaesthesiology, Pain & Palliative medicine, Radboud University Medical Centre, Nijmegen, The Netherlands

**Keywords:** Chronic obstructive pulmonary disease, Palliative care, Implementation, Evaluation studies

## Abstract

**Objectives:**

Little direction exists on how to effectively implement palliative care for patients with COPD. In the COMPASSION study, we developed, executed, and evaluated a multifaceted implementation strategy to improve the uptake of region-tailored palliative care intervention components into routine COPD care. We evaluated the implementation strategy and assessed the implementation process, barriers, and facilitators.

**Methods:**

A mixed methods process evaluation was performed. Primary and secondary healthcare providers in four hospital regions in the Netherlands were trained. Patients identified during hospitalisation for an acute exacerbation received palliative care and were followed for a year. Various sources were used: process data, questionnaires including the End-of-life Professional Caregiver Survey (EPCS), medical records, monitoring meetings, and interviews. The Consolidated Framework of Implementation Research (CFIR) was used to categorize implementation determinants.

**Results:**

The training sessions with roleplay were positively evaluated and increased professionals’ self-efficacy in providing palliative care statistically significantly. Of 98 patients identified, 44 (44.9%) received one or more palliative care conversations at the outpatient clinic. Having those conversations was highly valued by healthcare providers because it led to clarity and peace of mind for the patient and higher job satisfaction. Coordination and continuity remained suboptimal. Most important barriers to implementation were time constraints, the COVID-19 pandemic, and barriers related to transmural and interdisciplinary collaboration. Facilitators were the systematic screening of patients for palliative care needs, adapting to the patient’s readiness, conducting palliative care conversations with a pulmonologist and a COPD nurse together, and meeting regularly with a small team led by a dedicated implementation leader.

**Conclusions:**

Providing integrated palliative care for patients with COPD is highly valued by healthcare providers but remains challenging. Our findings will guide future implementation efforts. Future research should focus on how to optimize transmural and interdisciplinary collaboration.

Trial registration

The COMPASSION study is registered in the Netherlands Trial Register (NTR): NL7644. Registration date: 07/04/2019.

## Introduction

Patients with advanced Chronic Obstructive Pulmonary Disease (COPD) suffer from a high symptom burden and low quality of life, emphasizing the need for palliative care [1]. Palliative care is an approach that aims to optimize the quality of life of patients with a life-limiting illness through assessment and treatment of physical, psychological, social and spiritual problems [2]. It includes advance care planning, allowing care to be tailored to the patient’s goals and preferences [3]. Despite guideline recommendations, [4, 5] palliative care is only provided to a limited number of patients with COPD, and often, advance care planning is discussed in an acute care setting (e.g., when a patient visits the emergency department for an acute exacerbation) rather than proactively (e.g., during an outpatient visit to their regular doctor) [6, 7]. Also, the involvement of specialist palliative care is limited and restricted to the terminal phase [8]. As a result, many symptoms, such as dyspnoea, fatigue, and depression, remain undertreated, [9] and care preferences are not timely discussed [7].

Although the need for palliative care has been widely acknowledged, little direction exists on successfully implementing it into routine COPD care [10]. The key barriers to timely initiating palliative care in COPD are the prognostic uncertainty due to the unpredictable illness trajectory and the lack of training of healthcare providers (HCPs) to discuss end-of-life topics [11, 12]. These barriers may be addressed by using transition points, such as hospitalisation, to screen for palliative care needs [13] and communication training to increase HCPs’ self-efficacy in discussing palliative care topics [14]. However, the empirical evidence on effective implementation strategies is still limited [10].

Therefore, as part of the COMPASSION study, a multifaceted implementation strategy was developed, executed and evaluated [15]. HCPs across four hospital regions were trained to implement palliative care components into routine COPD care. Also, they were provided with access to an online toolbox, including a screening tool to identify palliative patients during hospitalisation, and implementation guidance. The aim of this study was 1) to evaluate the implementation strategy and its effect on reach and dose delivered of palliative care components and 2) to identify barriers and facilitators to successful implementation of integrated palliative care in COPD.

## Methods

### Design and setting

A comprehensive, mixed-method process evaluation was performed in four intervention hospital regions of the COMPASSION study. Each region was asked to form an intervention team consisting of primary and secondary care providers working in respiratory and palliative care (Table 1). We followed the Standards for Reporting Implementation Studies (StaRi) for reporting [16].

**Table 1:** Setting characteristics at baseline, indicators in the year before implementation and characteristics of the intervention team of each hospital region

	Region A	Region B	Region C	Region D
Characteristics of region				
Geographical setting	Large teaching hospital and surroundings	Regional hospital and surroundings	Regional hospital and surroundings	Regional hospital and surroundings
Pulmonologists / COPD nurses in hospital, n	6 / 4	5 / 2	5 / 4	5 / 3
COPD nurse(s) in primary care present	No	Yes	No	Yes
Protocol for PC in COPD present	No	No	No	No
Indicators in the year before implementation (2018)				
COPD patients hospitalised for acute exacerbation, n	367	149	143	220
Hospitalised patients with ≥1 specialised PC team consultation, n/n (%)	18/367 (4.9%)	4/149 (2.7%)	0/143 (0.0%)	24/220 (10.9%)
Characteristics of formed intervention team				
Total team members, n	11	10	9	16
Team composition, n				
Pulmonologists	2	3	2	4
COPD nurses in hospital	2	4	4	2
PC nurses in hospital	2	1	0	2
GPs	3	1	1	4
COPD nurses in primary care	0	1	0	2
PC nurses in primary care	0	0	0	2
Other	2	0	2	0
Implementation leader(s)	Pulmonologist + COPD nurse	Pulmonologist + 2 COPD nurses + PC nurse	COPD nurse + pulmonologist	COPD nurse + pulmonologist

Abbreviations: *COPD* chronic obstructive pulmonary disease, *GP* general practitioner; *PC* palliative care

### Intervention and implementation strategy

**Fig. 1:**
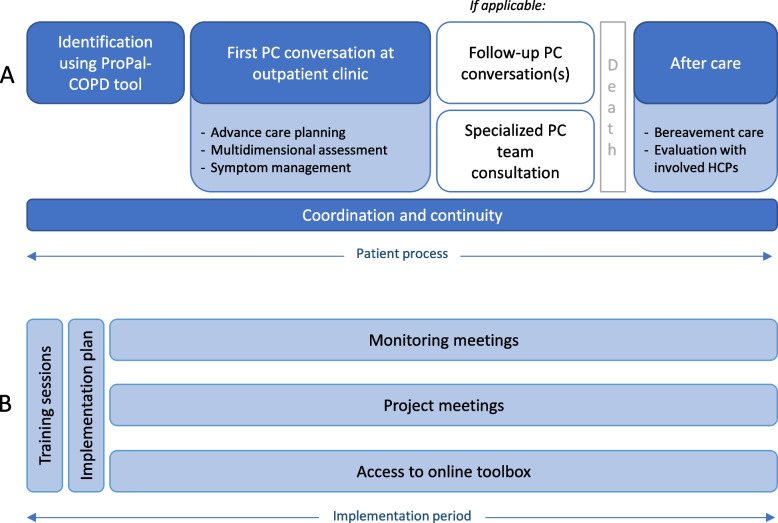
Components of the palliative care intervention (**A)** and implementation strategy (**B)**. HCP, healthcare provider; PC, palliative care

The intervention and multifaceted implementation strategy were developed in collaboration with many stakeholders and have previously been described in detail in the COMPASSION study protocol [15]. The intervention was based on national guidelines [2, 5] and consisted of the following core components (Fig. 1A): 1) identification of palliative patients with COPD during hospitalisation, 2) one or more palliative care conversations consisting of advance care planning, multidimensional assessment, and symptom management, 3) coordination and continuity of care, and 4) if a patient died, aftercare comprising bereavement care and care evaluation with involved HCPs. According to the national guideline, palliative care was performed primarily by respiratory HCPs, whereas specialist palliative care team consultants could be involved in case of complex needs [2]. Regions were allowed to tailor the intervention to regional and individual patients’ needs and preferences. The ProPal-COPD tool was used to facilitate providers to identify palliative patients admitted to the hospital for an exacerbation of COPD [17]. It consists of the surprise question (“Would you be surprised if your patient were to die in the next 12 months?”) and six COPD-specific clinical indicators, which together produce a total score. Initially, the cut-off value as previously published was used [17]. After six months, in monitoring meetings it became clear that the rate of positive scores was lower than expected by HCPs and researchers. Therefore, the research group deemed it necessary to lower the cut-off value.

To facilitate uptake of the intervention components, a multifaceted implementation strategy was developed (Fig. 1B). Between April and September 2019, HCPs from the intervention team received two 3-hour training sessions on 1) content of palliative care in COPD, including communication training with roleplay and non-pharmacological dyspnoea management, and 2) implementation of palliative care. At the end of the second training session, HCPs were asked to complete a regional action plan detailing how, when and by whom different intervention components had to be performed. They received access to an online toolbox (www.palliatievezorgcopd.nl), comprising information on the content and practice of the intervention components, the ProPal-COPD tool, and other tools for facultative use. Furthermore, implementation leaders were guided for two years through four monitoring meetings per region and two project meetings, where regions exchanged experiences and best practices.

### Data collection

The multifaceted implementation strategy was evaluated using process data (attendance rate of the training and use of regional action plans) and evaluation questionnaires administered at the end of each training session (appreciation of the training) and three and fifteen months later (use and appreciation of the toolbox). Also, HCPs’ level of self-efficacy in providing palliative care was assessed using the End-of-life Professional Caregiver Survey (EPCS) before and 3 and 15 months after the training [18]. This scale is a validated questionnaire comprising 28 items on three domains: patient- and family-centred communication, cultural and ethical values, and effective care delivery. The total score ranges from 0 to 112, with higher scores reflecting better knowledge and comfort in providing end-of-life communication.

To determine reach and dose delivered, we assessed the medical records of patients participating in the COMPASSION study one year after inclusion. Reach was defined as the number of patients participating in the intervention. Dose was defined as the extent to which each component was delivered [19]. Information on the number, timing, and content of palliative care conversations, treatments started, referrals made, and life-sustaining treatment preferences documented were extracted. For each included patient, HCPs were asked to indicate the duration of palliative care conversations, who was present, and the reason if no conversation had taken place.

Barriers and facilitators to implementation were identified using transcripts of monitoring meetings with implementation leaders (held by EV and JB) and semi-structured interviews with implementation leaders and trained HCPs less actively involved in implementation (held by JB). Between fifteen to twenty months after the training, per region, six HCPs (*n* = 24), were interviewed about care practices and work agreements, experiences with the implementation process, barriers and facilitators encountered, and experiences with the intervention. Interview duration varied between 20 and 85 minutes (mean 49 minutes). Monitoring meetings and interviews were audio-recorded and transcribed verbatim. All participants gave written informed consent, except for one GP due to time constraints, and this transcript was therefore excluded from analysis.

### Data analyses

Quantitative data were analysed using descriptive statistics with IMB SPSS (Statistical Package for Social Science) version 25. EPCS total scores were calculated, and pre-post scores of HCPs with complete EPCS data were compared using Wilcoxon signed rank tests. Qualitative data were analysed using thematic analysis with a phenomenological approach [20]. Transcripts were first inductively coded via open and axial coding (JB). Initial codes and summaries were discussed with the research group multiple times, and codes were merged, split, and renamed until consensus was reached. Subsequently, the Consolidated Framework of Implementation Research (CFIR) was used to further categorize possible barriers and facilitators to implementation [21]. The CFIR contains 39 constructs across five domains: intervention characteristics, outer setting, inner setting, characteristics of the individuals involved, and the process of implementation. Each code was mapped to one of the constructs by JB and checked by RK, who has extensive expertise in implementation research. Differences between and within regions were compared. Finally, the interpretation of findings was discussed with the research group.

## Results

### Evaluation of implementation strategy

The first and second training session attendance rates were 38/46 (82.6%) and 36/46 (78.3%), respectively. HCPs evaluated the first training session high with a mean score of 8.4 out of 10 and the second training session with 7.9. The highest rated training elements were interactive communication training and dyspnoea management. After three months, 18/29 responding providers (62.1%) reported to have visited the online toolbox at least once, and 20/28 responding providers (71.4%) after fifteen months. The online toolbox was evaluated with a score of 7.1 for design and 7.8 for content. A draft of a regional action plan was completed by two regions, but not actively used in practice.

A statistically significant increase in EPCS total scores was observed three and fifteen months after the training (Table 2).

**Table 2:** Comparison of healthcare provider’s End-of-life Professional Caregiver Survey (EPCS) scores at baseline and 3 and 15 months after the training

	Number of complete cases	Median score	*Z*^*a*^	*p*-value
Before training (baseline)	37	81.0		
After 3 months	26	89.5	4.03	<.001
After 15 months	23	92.0	3.44	.001

^a^Wilcoxon signed rank test

### Reach and dose of intervention components

#### Component 1. Identification

All 198 hospitalised patients eligible and consenting to participate in the COMPASSION study (100%) were screened with the ProPal-COPD tool (Table 3). Of these, 98 had a positive ProPal-score. HCPs also screened outpatients on their initiative, but as these patients did not participate in the COMPASSION study, they were not included in the numbers.

**Table 3:** Reach and dose of palliative care intervention components per region

Component		Region A	Region B	Region C	Region D	Total
1. Identification	Number of hospitalised patients screened using ProPal-COPD tool	48	73	45	32	198
	Number of patients with positive ProPal-score	29	31	17	21	98
2. Palliative care conversations	Patients who received ≥1 outpatient palliative care conversation	15/29	11/31	10/17	8/21	44/98
	Patients who received ≥1 specialist palliative care team consultation	3/29	14/31	0/17	5/21	22/98
3. Coordination and continuity	Number of letters to GP following an outpatient palliative care conversation	13/15	7/11	0/10	2/8	22/44
	Individual care plan	0/15	0/11	0/10	0/8	0/44
4. Aftercare	Conversation with bereaved family of deceased patients	0/7	0/9	0/1	0/4	0/21
	Evaluation by involved healthcare providers	0/7	0/9	0/1	1/4	1/21

#### Component 2. Palliative care conversations

A palliative care conversation at the outpatient clinic within 1-year follow-up occurred in 44/98 patients with a positive ProPal-score (44.9%). The timing, duration, and content of the conversations are presented in Table 4. In some cases, a conversation was waived due to organisational factors: transferral to a different care setting (primary care, rehabilitation centre, or nursing home) (*n* = 9) or postponement of outpatient visits due to the COVID-19 pandemic (*n* = 6). In other cases, the patient had died (n = 9), was reluctant (*n* = 7), or was psychiatrically ill (*n* = 1).

**Table 4:** Timing, duration and content of 61 outpatient palliative care conversations in 44 patients

	Findings
Palliative care conversations	
Timing of first palliative care conversation	Median 42 days (IQR 24.25–96.25) after inclusion 33/44 (75.0%) within 3 months 36/44 (81.8%) within 6 months
Average duration	Region A: 60 minutes Region B: 60 minutes Region C: 30 minutes Region D: 15 to 30 minutes
Number of conversations per patient	
1	32 (72.7%)
2	7 (15.9%)
3	5 (11.4%)
Healthcare providers present	Pulmonologist + COPD nurse: 32x (52.5%)
	Pulmonologist: 6x (9.8%)
	COPD nurse: 23x (37.7%)
Advance care planning	
Topics discussed	
Illness trajectory	19 (43.2%)
Incurability of the disease	7 (15.9%)
Life expectation	11 (25.0%)
Care goals	9 (20.5%)
Advantages and disadvantages of life-sustaining treatment	23 (52.3%)
Preferences for hospitalisation in case of a next exacerbation	16 (36.4%)
Preferred place of death	8 (18.2%)
Palliative sedation and/or euthanasia	10 (22.7%)
Documentation of life-sustaining treatment preferences	34 (77.3%)
Multidimensional assessment	
Domains addressed	
Physical	43 (97.7%)
Psychological	30 (68.2%)
Social	33 (75.0%)
Spiritual	27 (61.4%)
Symptom management	
Breathlessness treated with opioids	19 (43.2%)
Non-pharmacological breathlessness interventions	26 (59.1%)
Advice and breathing techniques	15
Oxygen therapy	11
Handheld fan	7
COPD action plan	2
Treatment for anxiety and depression	11 (25.0%)
Pharmacological treatment	7
Referral to psychologist	5
Breathing techniques	2
Involving palliative care nurse	1
Patients referred	32 (72.3%)
Physiotherapist	21
Tertiary pulmonary rehabilitation	12
Primary care COPD nurse	11
Psychologist	5
Dietician	5
Occupational therapist	2
Primary care palliative care nurse	2
Spiritual counsellor	1

Twenty-two of 98 patients (22.4%) received a specialist palliative care team consultation and were subsequently discussed in the multidisciplinary team meeting; the percentage varied between regions from 0 to 45% (Table 3).

#### Component 3. Coordination and continuity

In half (22/44) of patients receiving an outpatient palliative care conversation, a letter was sent to the GP to report the conversation, in which nine agreements about future care coordination were included. None of the regions noted creating an individual care plan.

#### Component 4. Aftercare

Of all 98 patients, 21 patients died within one year of follow-up. An aftercare conversation was occasionally offered to bereaved families but never occurred in practice, and an evaluation of HCPs involved took place once.

### Barriers and facilitators to successful implementation

Characteristics of interview participants are described in Table 5. For each domain of the CFIR, the facilitators and barriers identified are summarised in Table 6. In the outer setting domain, no determinants were identified.

**Table 5:** Characteristics of interview participants (*n* = 24)

Characteristic	Value
Mean age in years ±SD (range)	49.8 ± 9.6 (33–62)
Age category, n(%)	
30–39	4 (16.7)
40–49	7 (29.2)
50–59	9 (37.5)
60–69	4 (16.7)
Female sex, n(%)	18 (75.0)
Profession, n(%)	
Pulmonologist	7 (29.2)
COPD nurse	9 (37.5)
General practitioner	4 (16.7)
Palliative care nurse	4 (16.7)
Years in profession, mean ± SD (range)	10.9 ± 9.3 (1–32)
Years in profession, n(%)	
< 5	7 (29.2)
5–10	8 (33.3)
≥10	9 (37.5)

**Table 6:** Facilitators (F) and barriers (B) that affected the process of implementing palliative care into regular COPD care

Domain	Constructs	F/B	Main findings
Intervention characteristics	Relative advantage	F	The intervention was highly valued because it provided clarity, peace of mind, and less anxiety to the patient, improved the relationship with the patient, and increased job satisfaction.
		F	Systematic screening of patients helped HCPs to become aware of palliative care needs.
	Perceived difficulties of the intervention	B	Patients responded differently to the intervention. It was relieving for some and it was confronting for others. It was considered essential to adapt to the patient’s level of readiness.
		B	All HCPs felt that transmural collaboration was still inadequate. Raised issues were: challenge to have phone contact due to busy schedules, lack of an appropriate communication tool, doubt about what and how to communicate, and lack of COPD nurses in primary care.
Inner setting	Tension for change	F	Almost all HCPs believed that (better structured) palliative care for patients with COPD was highly necessary.
		B	Two HCPs found that they already did many things well and that change was not needed.
	Available resources	B	Due to busy schedules, it was challenging to schedule palliative care conversations.
	Networks and communications	B	The division of roles between HCPs of the pulmonary department and the specialist palliative care team was unclear.
	Relative priority	B	The COVID-19 pandemic caused changed priorities, resulting in the postponement of palliative care conversations.
Characteristics of individuals	Knowledge and beliefs about the intervention	F	The observed positive effects on patients motivated to continue with the intervention.
		F	Sharing experiences in implementing and organizing integrated palliative care between regions was considered useful and inspiring to continue the intervention.
	Self-efficacy	F	Communication training and being provided with example phrases were perceived as helpful.
		F	Conducting palliative care conversations (in part) together with a COPD nurse helped the pulmonologist to discuss non-medical topics and it saved time.
Implementation process	Planning	B	HCPs found it challenging to formulate clear implementation goals and to plan actions.
	Reflecting & Evaluating	F	Regular meetings with a small team helped to make implementation agreements.
	Engaging	B	Implementation was primarily focused on planning palliative care conversations in the outpatient clinical setting. Team members from primary care and palliative care were not actively involved in the implementation process because their potential role was unclear.
	Implementation leaders	F	A dedicated implementation leader feeling responsible for the implementation was essential.
		B	Without someone explicitly made responsible, implementation was hampered.

Abbreviations: *HCP* healthcare provider

### Intervention characteristics

#### Relative advantage

All HCPs highly appreciated the palliative care intervention because its implementation resulted in more clarity and peace of mind for patients, improved the relationship with patients, and provided HCPs with a sense of contributing in a meaningful way.

*“It also gives a lot of satisfaction in your work, that you can help people in that way too, those patients. So I also personally really enjoy it because of that.” Pulmonologist 6.*

Pulmonologists and COPD nurses across all regions indicated that systematic screening of patients had enhanced their awareness of palliative care needs.

*“[…] in the past, I often thought, oh, it’ll be fine, he’ll still have years. And now I’m more alert to it, so I think that’s a really important factor, which makes me think more quickly that we need to have a conversation.” Pulmonologist 2.*

#### Perceived difficulties of the intervention

HCPs across all regions experienced that most patients were open to discussing palliative care topics. However, reactions differed, and adapting to the patient’s level of readiness was found essential.

*“Um, at the beginning of the project, I did it quite abruptly […]. I also noticed that people were a bit frightened, [...] that I thought, oh yes, this has to be done more gradually.” COPD nurse 7.*

Across all regions, the collaboration between the hospital and primary care was perceived inadequate due to time constraints and lack of an appropriate communication tool. Also, some pulmonologists had doubts about what to communicate to GPs, as the level of palliative care expertise varied greatly between GPs. COPD nurses in primary care were found to be important for adequate transmural communication, but they were not always available due to staff shortages and budget cuts.

### Inner setting

#### Tension for change

Almost all HCPs felt that improvement in palliative care was highly needed and they were willing to improve care.

#### Available resources

Busy schedules made planning palliative care conversations challenging, particularly when both a pulmonologist and COPD nurse were involved. In one region, this was solved by reserving a weekly set time in the pulmonologist’s agenda. Whether conversations were scheduled depended greatly on clear working arrangements and staff continuity.

#### Relative priority

When the COVID-19 pandemic broke out in March 2019, HCPs experienced that priorities changed. Multidisciplinary meetings were cancelled, and palliative care conversations were postponed to keep patients out of the hospital.

#### Networks and communications

In each region, a COPD nurse became part of the specialist palliative care team to exchange knowledge. However, the extent of and satisfaction with collaboration between pulmonary and palliative care providers varied between regions. In one region, friction arose because palliative care providers had expected to become involved more often, but pulmonary care providers found them too direct in their approach to patients with COPD.

### Characteristics of the individuals

#### Knowledge and beliefs about the intervention

Experiencing the positive effects on the patient motivated HCPs across all regions to continue implementing the intervention.

*“Because you do the questionnaire* [ProPal-score] *with the patient, is it positive or not? And you also schedule appointments with the patient in a really clear way, it gives it all structure and clarity and by doing it you gain self-confidence and the reaction of the patient is generally very positive and yes, that also gives us a reason to continue, well, the way we took is just the right way.” COPD nurse 5.*

Also, sharing experiences with other regions during the project meetings was reported by four HCPs to be very helpful.

#### Self-efficacy

Most pulmonologists and two COPD nurses reported initially feeling uncomfortable starting a palliative care conversation, but their confidence increased the more they did it. The communication training and example phrases were perceived as helpful. Most pulmonologists and COPD nurses preferred to hold the conversations partly together because it was more efficient and made it easier to start the conversation.

*“That actually really helped me, I think, it also supported me a bit, that I found it a little less scary. Because it is quite difficult to start a conversation like that.” Pulmonologist 2.*

*“Because […] I do the introductory talk, it’s easier for the pulmonologist to continue the conversation in that half hour. Um, and in this way it’s a bit more structured, the pulmonologist doesn’t have to block a full hour for it, and in this way, we complement each other well.” COPD nurse 2.*

### Implementation process

#### Planning, Reflecting and Evaluating

Regional action plans were not used to guide implementation, but HCPs of three regions indicated that they made verbal work agreements. Working together in a small team helped to make those agreements and keep them. HCPs of one region noticed that it worked well to schedule weekly meetings at a fixed time.

*“The big stick is that you just get together every week, [...] to implement the actions that each person is assigned.” COPD nurse 2.*

#### Engaging

In all regions, implementation was primarily focused on identification and palliative care conversations. As a result, transmural collaboration only came into focus later in the project. To the disappointment of some, team members from primary care and specialist palliative care were not actively involved in the implementation process because their potential contribution was unclear.

*“I didn’t notice so much here the role of the specialised general practitioner. I had a different expectation.” General Practitioner 2.*

#### Implementation leader

A dedicated implementation leader feeling responsible for the implementation and keeping everyone engaged was deemed essential by HCPs across all regions. In one region, no one was explicitly made responsible, which hampered implementation.

## Discussion

### Main findings

This mixed-method study provides a detailed understanding of the implementation process of palliative care components into routine COPD care, how a multifaceted strategy can influence this process, and essential factors for successful implementation. Training sessions with roleplay were positively evaluated and increased the self-efficacy in providing palliative care. Of all patients screened, around half received an outpatient palliative care conversation, on average six weeks after inclusion and mostly held by a pulmonologist and COPD nurse together. Continuity and coordination of care remained limited, and aftercare was not done at all. The most important implementation barriers were time constraints, the COVID-19 pandemic, and barriers related to interdisciplinary and transmural collaboration. Factors facilitating implementation were: the systematic screening of palliative patients, adapting to the patient’s readiness, conducting palliative care conversations together with a pulmonologist and COPD nurse, and meeting regularly with a small team led by a dedicated implementation leader. Our findings will guide future implementation efforts to integrate a palliative care approach into COPD care and provide insights into the most effective components.

### Implementation strategies

A multifaceted implementation strategy was used to optimize uptake of the intervention, [22] but the appropriateness varied per individual strategy. In line with previous research, communication training with roleplay by actors was positively evaluated by HCPs and increased their self-efficacy in providing palliative care [14, 23, 24]. Also, sharing best practices between regions during project meetings was positively evaluated and perceived as inspiring to continue implementation. However, the online toolbox and regional action plans were less used than anticipated. Filling in the plans proved too abstract and time-consuming for busy HCPs. As a result, implementation proceeded largely unstructured and depended greatly on the implementation leader’s enthusiasm. For future implementation efforts, we recommend providing HCPs with clear instructions and practical ready-to-use tools and scheduling frequent team meetings led by a dedicated implementation leader.

### Palliative care conversations

Systematic screening of patients appeared to be an essential intervention component. It raised HCPs’ awareness and made them more ready to initiating palliative care conversations. However, the ProPal-COPD tool’s performance appeared to be unsatisfactory. External validation results and user experiences will be discussed in a separate publication. With 45% of patients identified, a palliative care conversation was held. Despite of the COVID-19 pandemic, this percentage is comparable to previous studies. In the systematic review of Houben et al. on advance care planning interventions, [25] the occurrence of palliative care conversations in intervention groups of included studies ranged from 18 to 64% [26–30]. HCPs were very positive about the palliative care discussions, but alignment with patient readiness was deemed important as COPD is not considered as ‘potentially lethal’ by most patients [31]. It is less confronting to patients if advance care planning is initiated gradually with topics related to dying and death introduced step-by-step over multiple conversations. In our study, using a dual-track approach (“hope for the best, and prepare for the worst”), [32] it was possible to introduce such topics already in an earlier stage. Pulmonologists highly valued collaboration with a COPD nurse as it helped them discuss sensitive topics and saved time. Indeed, blocking enough time for the palliative care conversations was challenging. Therefore, scheduling conversations at the end of the consultation hour to allow for possible extension or scheduling a fixed time in the week is recommended.

### Interdisciplinary collaboration

In line with guideline recommendations and care practices in the Netherlands, [4, 5, 33, 34] our intervention was delivered by respiratory HCPs (so-called generalist care providers), while specialist palliative care providers were only involved in the case of complex care needs. In our study, the level of involvement varied across regions. Respiratory HCPs were reluctant to involve the specialist palliative care team because they lacked COPD-specific knowledge regarding treatment and communication practices. Specialist palliative care providers are mainly involved with oncology patients, [35] whereas patients with COPD require a different approach [36]. Therefore, it should be further explored how respiratory and palliative care HCPs optimally collaborate and learn from each other’s expertise.

### Transmural collaboration

The intervention component coordination & continuity was less well implemented across all regions. Although providers from primary care and the hospital were involved in the training, implementation leaders first focused on organizing outpatient palliative care conversations. Consequently, transmural collaboration received insufficient attention, as reflected by the low number of letters sent from hospital to the GP. Although HCPs expressed that contact by phone is preferred to optimize care coordination and continuity after a patient was identified, this was not always done due to time constraints and lack of a shared electronic medical record. Therefore, a communication tool to facilitate bidirectional communication (ideally digital, linked to medical files, and always accessible) is needed. Further, COPD nurses in primary care play an essential role in linking primary and secondary care and should be available in every region. Moreover, to optimize coordination and guarantee continuity of care, financial structures that allow flexibility and ‘shared care’ are warranted.

### Strengths & limitations

This is the first comprehensive study assessing palliative care implementation in a real-world outpatient COPD care setting. We used different data sources to provide a broad and in-depth understanding of the implementation process. Furthermore, the intervention and implementation strategy were designed using theory, guidelines, and input of many stakeholders, ensuring that barriers from the field were addressed [15]. However, our study also has some limitations. First, the COVID-19 pandemic had severe implications that may have biased our findings. HCPs had less time for implementation activities, multidisciplinary meetings were put on hold, and palliative care conversations were cancelled to prevent contamination. Second, our implementation results were somewhat constrained because it was performed alongside a cluster randomised controlled trial (as part of a hybrid type 2 effectiveness-implementation study) [15]. Next to the positive aspects of combining these two study objectives, such as faster knowledge development, [37] it limited our flexibility to adapt to new insights that emerged during the study. For example, the fixed inclusion criteria required for effectiveness evaluation limited the measured reach because palliative patients identified at the outpatient clinic could not be included. Also, HCPs were focused on enrolling patients for sufficient power of the effectiveness study, limiting their time for implementation activities. Finally, we did not assess the quality of implementation, e.g. the quality of palliative care conversations. In future studies, this could be assessed using conversation analysis, as was found to be a viable method by Otte et al. [38].

### Conclusion

Implementation is a complex process, and dedicated action is needed to ensure theoretically promising and highly needed interventions, such as palliative care for patients with COPD, are delivered as intended. The multifaceted implementation strategy evaluated in the COMPASSION study demonstrated the importance of communication training in discussing palliative care topics with patients with COPD in a sensitive way, systematic screening of patients with palliative care needs, and a structured implementation process led by a dedicated implementation leader. It also highlighted that we are not there yet; future research should focus on optimizing transmural and interdisciplinary collaboration, to ensure optimal integration and continuity of palliative COPD care.

## Data Availability

The datasets generated and analysed during the current study are not publicly available due to the confidentiality and the traceability of the data but are available from the corresponding author on reasonable request.

## Abbreviations

CFIR: Consolidated Framework of Implementation Research
COPD: chronic obstructive pulmonary disease
EPCS: End-of-life Professional Caregiver Survey
HCP: healthcare provider
